# Supplementary material for: Natural Products Extracted from Fungal Species as New Potential Anti-Cancer Drugs: A Structure-Based Drug Repurposing Approach Targeting HDAC7
Source: Molecules. 2020 Nov 25;25(23):5524. doi: 10.3390/molecules25235524 (PMC7728054; doi:10.3390/molecules25235524)

Supplementary Material for

Natural products extracted from fungal species as new potential anti-cancer drugs: a structure-based drug repurposing approach targeting HDAC7

Annalisa Maruca [1,2#], Roberta Rocca [2,3#], Raffaella Catalano [1,2], Francesco Mesiti [1,2], Giosuè Costa [1,2], Delia Lanzillotta [3], Alessandro Salatino [3], Francesco Ortuso [1,2], Francesco Trapasso [3], Stefano Alcaro [1,2*], Anna Artese [1,2]

[1]Dipartimento di Scienze della Salute, Università "Magna Græcia" di Catanzaro, Campus "S. Venuta", Viale Europa, 88100, Catanzaro, Italy.
[2]Net4Science Academic Spin-Off, Università "Magna Græcia" di Catanzaro, Campus "S. Venuta", Viale Europa, 88100, Catanzaro, Italy.
[3]Dipartimento di Medicina Sperimentale e Clinica, Università "Magna Græcia" di Catanzaro, Campus "S. Venuta", Viale Europa, 88100, Catanzaro, Italy.

* Correspondence: alcaro@unicz.it; Tel. +39 0961 3694198 (S. A.)

#These authors contributed equally.

Table of Contents

Figure S1. 3D and 2D representations of the best re-docking pose of Trichostatin A (TSA) against HDAC7 (PDB code 3C10) receptor.
Figure S2. 3D and 2D representations of the **(R)-2** complexed to HDAC7.
Table S1. Name, 2D structure and G-Score value (kcal/mol) of the **(R)-2**.
Figure S3. Root Mean Square Deviation trends of both HDAC7 and ligands heavy atoms in presence of *hit* **(S)-2** and TSA.
Figure S4. Root Mean Square Fluctuation trend of HDAC7 residues in presence of *hit* **(S)-2** and TSA, and secondary structure elements (SSE) distribution of both TSA and *hit* **(S)-2**.
Figure S5. Ligand RMSF trend of both *hit* **(S)-2** and TSA.

Figure S1. **(a)** 3D representation of the best re-docking pose of Trichostatin A (TSA) against HDAC7 (PDB code 3C10) receptor obtained using the Glide-SP algorithm. The protein is shown as grey surface, while the amino acid residues involved in the molecular interactions are reported as grey carbon sticks. Re-docked and crystallographic conformations of TSA are displayed as green and orange carbon ball-and-sticks, respectively. **(b)** 2D representation of the key interactions of TSA in the binding pocket of HDAC7, according to the crystallographic pose.

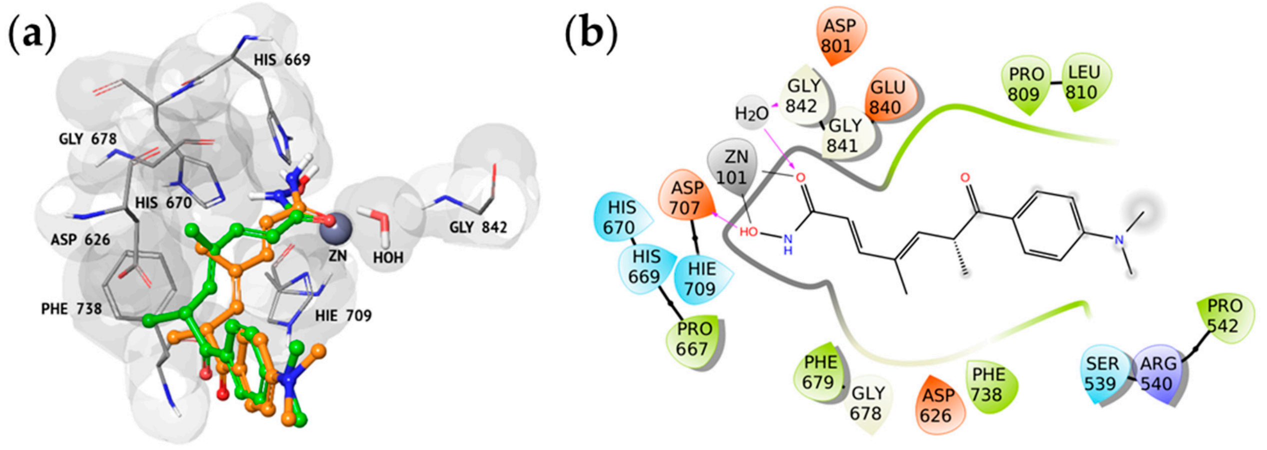

Figure S2. **(a)** 3D representations of the **(R)-2** complexed to HDAC7. The protein is shown as grey surface, the ligands are displayed as green carbon ball-and-sticks, while the amino acid residues involved in the molecular interactions are reported as grey carbon sticks. **(b)** 2D representation of the key interactions of the **(R)-2** in the binding pocket of HDAC7.

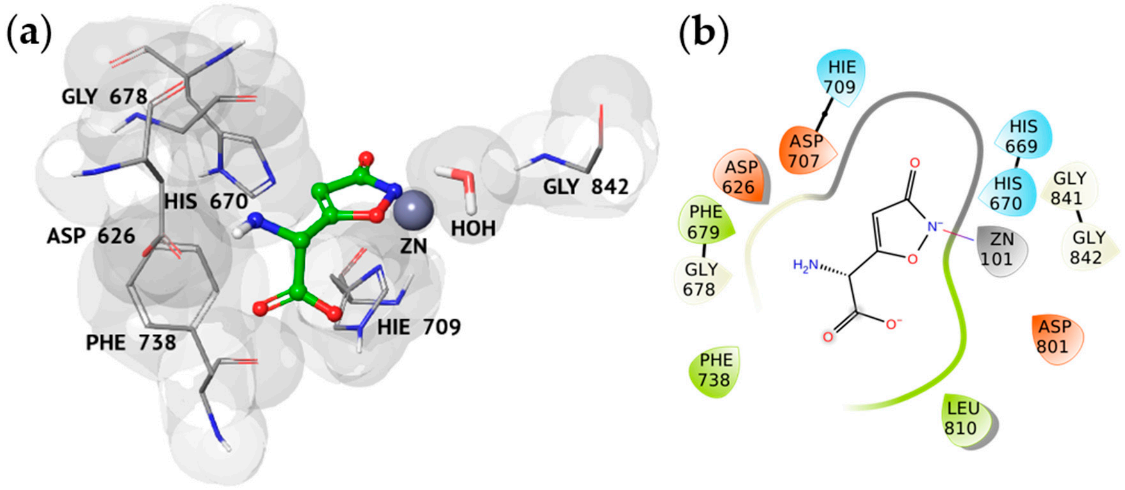

Table S1. Name, 2D structure, and G-Score value (kcal/mol) of the **(R)-2**.

Name	2D Structure	G-Score *(kcal/mol)*
**(R)-2** or (R)-Ibotenic acid	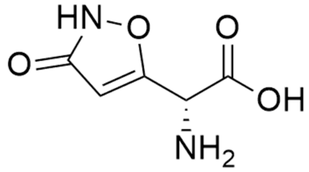	-6.50

Figure S3. (a) Root Mean Square Deviation (RMSD) trend of HDAC7 heavy atom in presence of *hit* **(S)-2** and TSA, indicated by a red and a green line, respectively. **(b)** RMSD trends of ligands heavy atoms, after the alignment of the complex on the protein backbone of the first MD frame structure.

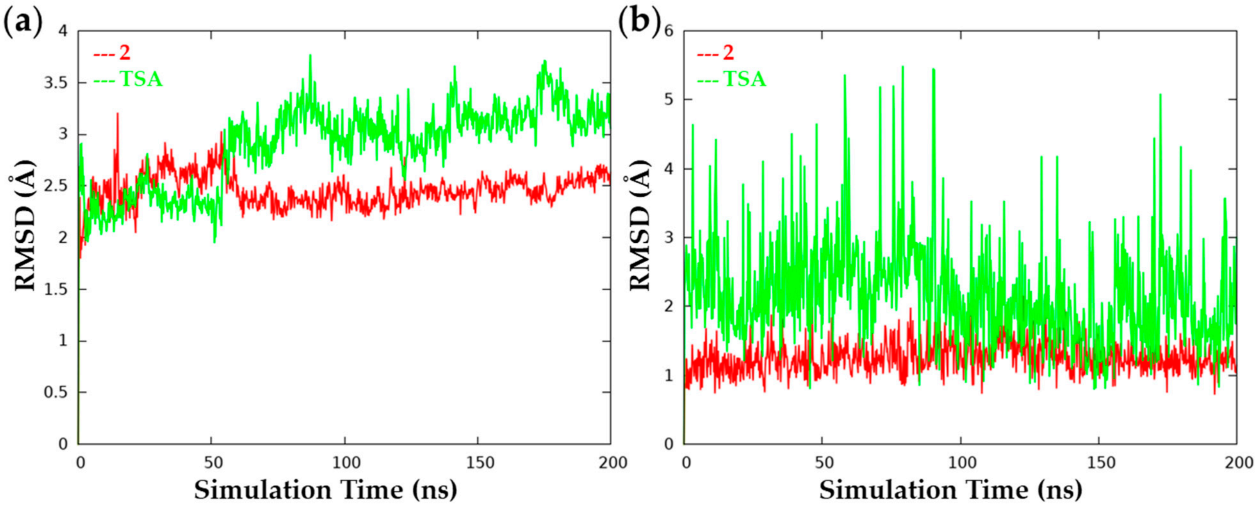

Figure S4. (a) Root Mean Square Fluctuation (RMSF) trend of HDAC7 residues in presence of *hit* **(S)-2** and TSA, indicated by a red and a green line, respectively. Secondary structure elements (SSE) distribution by residue index of: **(b)** TSA and **(c)** *hit* **(S)-2** throughout the protein structure during the whole simulation. Protein SSE like alpha-helices and beta-strands are represented in orange and cyan Gaussian curves, respectively.

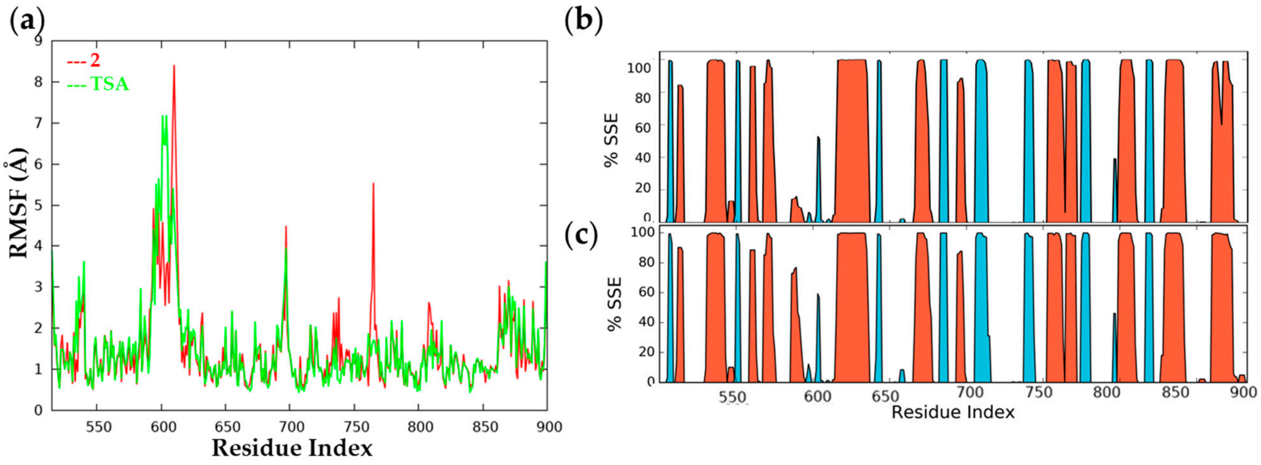

Figure S5. (a) Ligand RMSF trend of *hit* **(S)-2**. **(b)** Ligand RMSF trend of TSA. RMSF shows the ligand's fluctuations broken down by atom, corresponding to the 2D structure in the top panel. The protein-ligand complex is first aligned on the protein backbone and then the ligand RMSF is measured on the ligand heavy atoms.

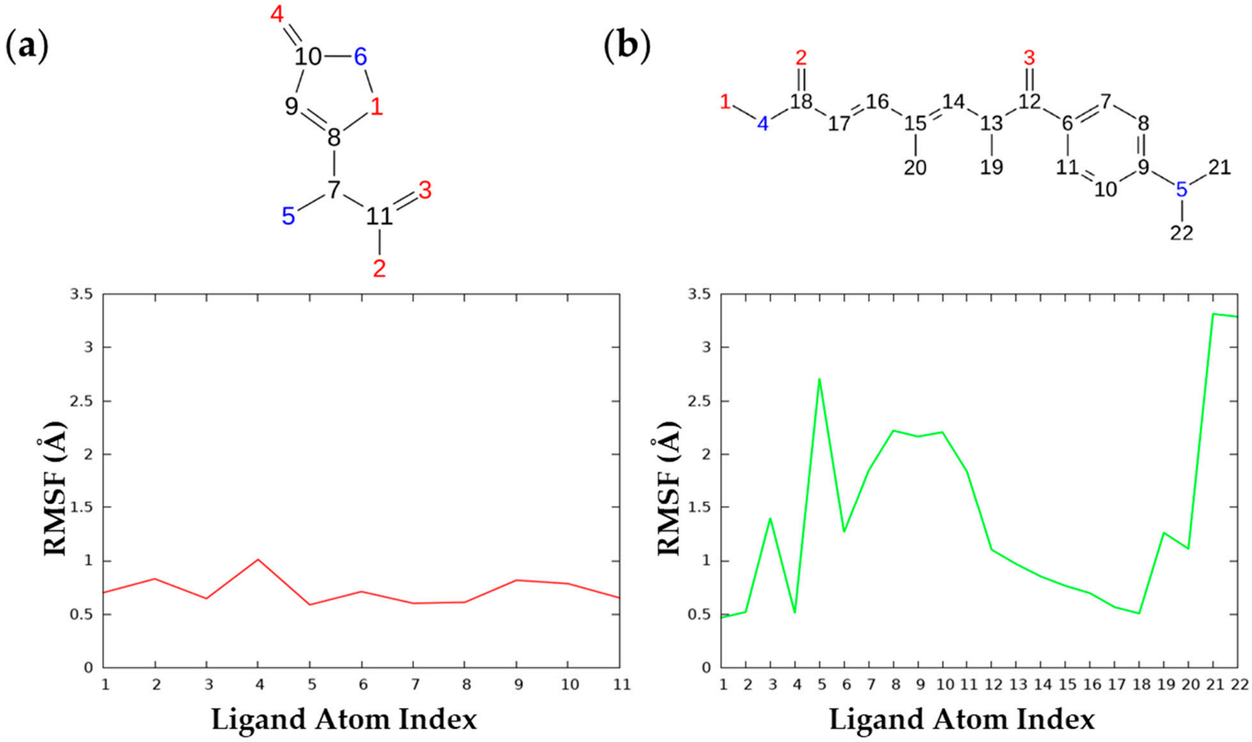

Supplement: Supplementary file 1 [file molecules-25-05524-s001.pdf]
